# The sense and nonsense of antipsychotic combinations: A model for dopamine D_2/3_ receptor occupancy

**DOI:** 10.1038/s41398-025-03582-2

**Published:** 2025-10-01

**Authors:** Moritz Spangemacher, Christian N. Schmitz, Paul Cumming, Luca V. Färber, Xenia M. Hart, Hiroyuki Uchida, Gerhard Gründer

**Affiliations:** 1 Department of Molecular Neuroimaging, Central Institute of Mental Health, Medical Faculty Mannheim, University of Heidelberg, Mannheim, Germany; 2 Central Institute of Mental Health, Department of Psychiatry, Medical Faculty Mannheim, University of Heidelberg, Mannheim, Germany; 3 German Center for Mental Health (DZPG), partner site Mannheim-Heidelberg-Ulm, Mannheim, Germany; 4 Department of Nuclear Medicine, Bern University Hospital, Bern, Switzerland; 5 School of Psychology and Counselling, Queensland University of Technology, Brisbane, Australia; 6 Department of Neuropsychiatry, Keio University School of Medicine, Tokyo, Japan

**Keywords:** Molecular neuroscience, Schizophrenia

## Abstract

Approximately 20–30% of patients treated for schizophrenia concomitantly take two or more antipsychotic substances, despite the limited evidence that antipsychotic combination treatment is superior to monotherapy. Positron emission tomography (PET) studies can reveal the relationship between plasma levels of an antipsychotic medication and occupancy at striatal dopamine D_2/3_ receptors (D_2_R), but there is scant consideration in the literature of the net occupancy obtained with antipsychotic combination treatment. In this report, we introduce a novel model for predicting net D_2_R occupancy in antipsychotic polypharmacy (APP); taking as illustrative examples five commonly prescribed antipsychotic medications. In an extension of the law of mass action for predicting receptor occupancy from the plasma concentration of a single psychopharmacological agent, we test a model for inferring the net striatal D_2_R occupancy in APP from the individual Michaelis-Menten kinetics of two (or more) antipsychotic medications. Based on literature PET findings for striatal D_2_R occupancy in monotherapy, our model predicts that widely used antipsychotic medication combinations may exceed the optimal therapeutic window of 65–80% occupancy. Our extended model accurately predicted occupancy for the only APP combination documented by PET. Present results call for caution in the design of antipsychotic medication combination therapy, aiming to avoid excessive occupancy by adjusting drug concentrations and doses.

## Introduction

Antipsychotic polypharmacy (APP), which entails the administration of two or more antipsychotic medications, is a common clinical practice in the treatment of schizophrenia [1, 2]. An analysis of 147 clinical studies indicated that approximately 20 – 30% of all patients treated with antipsychotics take more than one antipsychotic medication [3]. Although most international schizophrenia guidelines specifically recommend against APP, it remains prevalent worldwide [4–6].

Treatment with APP most frequently arises from a need to enhance antipsychotic efficacy in the context of treatment-resistant schizophrenia [7]. However, there is a general lack of clear evidence supporting the benefits of antipsychotic combination treatment. Thus, a Cochrane review of sixty-two randomized studies comparing APP with antipsychotic monotherapy for the treatment of schizophrenia and/or schizophrenia-like psychoses found “very low-quality evidence that a combination of antipsychotics may improve the clinical response“ [8]. Furthermore, the authors of the review did not find APP to be superior in preventing relapse, reducing the risk of premature discontinuation of the treatment, or regarding the risks of hospitalization or serious adverse events as compared to antipsychotic monotherapy [8]. Another meta-analysis likewise supported the general non-superiority of APP, but did indicate improvement of negative symptoms upon augmentation of monotherapy with a dopamine D_2/3_ receptor (D_2_R) partial agonist [9]. Furthermore, there is some suggestion that APP may to be associated with lesser side effects as compared to monotherapy [7]. Individual combinations of antipsychotic medications differ with respect to the likelihood and type of adverse events [10]. Due to their differing pharmacodynamic and pharmacokinetic profiles, side effects vary widely between different antipsychotic agents, which raises the possibility that oposing actions at D_2_Rs could lead to diminished side effects, or conversely that additive effects could result in exacerbation of side effects. However, there is a lack of clear evidence for such interactions arising in the context of APP [11, 12].

In contrast to the results of the Cochrane review, national survey studies in Finland showed that APP could reduce the risk of psychiatric and all-cause re-hospitalization, suggesting that APP might indeed offer long-term benefits, at least within certain patient subgroups [13]. However, results of a prospective clinical trial did not indicate superiority of APP over antipsychotic monotherapy with respect to long-term outcome [14]. In summary, there is little evidence supporting the use of APP in the clinical context. Furthermore, physicians may resort to APP without having exhausted the possibilities of antipsychotic monotherapy [15], or having given a trial with evidence-based treatments for treatment-resistant schizophrenia such as clozapine [16, 17] or electroconvulsive therapy [18].

The common neurobiological rationale that combination therapy engages a broader range of pharmacodynamic targets may fail to consider the need for dose adjustment, which could become necessary due to additive occupancy at dopamine D_2_Rs in the brain [19]. All available antipsychotics exert their main antipsychotic actions through antagonism or partial agonism at D_2_R [20–22], but these same receptors also mediate psychomotor side effects [23] as well as sexual and endocrine dysfunctions such as hyperprolactinemia [24, 25]. Furthermore, high antagonist occupancy at D_2_R is a factor in more severe adverse events, i.e., antipsychotic-induced catatonia and neuroleptic malignant syndrome [26, 27].

The need to assess target engagement at D_2_R motivated an extensive body of research using selective radioligands for positron emission tomography (PET) or single photon emission computed tomography (SPECT). A compilation of such studies supports the consensus that effective relief of positive symptoms requires D_2_R antagonist occupancy in the range of 65–80% [28, 29], whereas extrapyramidal side effects can beome problematic and occur more frequently with occupancies in excess of 80% [30]. This “therapeutic window” of 65–80% receptor occupancy applies to most of the second-generation antipsychotics, excepting the low-affinity D_2_R antagonists such as clozapine and quetiapine, which tend to obtain therapeutic occupancies less than 65% [28]. On the other hand, partial dopamine D_2_R agonists like aripiprazole require a target engagement of at least 90% [31].

For most antipsychotic medications, the occupancy at brain dopamine D_2/3_ receptors bears a predictable relation to the plasma drug level [32]. However, there are scant reports of PET studies in the context of APP, other than a preclinical investigation in relation to effects of antipsychotic combinations on glucose tolerance in rats [33]. In the only net occupancy study, augmentation of clozapine monotherapy with a moderate dose of haloperidol (4 mg/day for 4–8 weeks) substantially increased the mean striatal D_2_R occupancy from 55 to 79% in a group of five patients with schizophrenia [34]. Given the high prevalence of various permutations of APP, there is inadequate knowledge about its net effects on occupancy. To address this issue, we developed in the present study a modelling approach for predicting net D_2_R occupancy in relation to APP. We tested out this model for the cases of five widely used combinations of antipsychotic medications.

## Materials and methods

### Computed model

We base our theoretical model on striatal D_2_R occupancy data derived from previously published PET studies [28]. To determine receptor occupancy by a given pharmaceutical, one measures the displacement of a selective radiotracer relative to specific binding in an unblocked baseline condition [35]. The maximum attainable receptor occupancy (E_max_) represents the percentage of receptors that can be occupied by a pharmaceutical, whereas the 50% “effective concentration” (EC_50_) represents the plasma concentration associated with half-maximal occupancy [31].

According to Michaelis-Menten principles for mass action, the receptor occupancy of a pharmacological compound can be determined as:

1 $${\rm{occupancy}}\,[ \% ]=({{\rm{E}}}_{\max }{\rm{x}}\,[{\rm{C}}])/({{\rm{EC}}}_{50}+[{\rm{C}}])$$

with [C] representing the plasma concentration of the drug. For the modelling of APP, we consider the occupancies of two (or more) different pharmaceutical compounds of different plasma concentrations and target affinities, with appropriate adaptation of the law of mass action. Here we assume that the several ligands bind independently at the same receptor population in a competitive manner, in consideration of the equilibrium concentrations of the receptor-ligand complexes and the free receptor, which together comprise the total number of available receptors:

2 $${{\rm{E}}}_{\max }={\rm{UR}}+{\rm{O}}1+{\rm{O}}2$$

where UR is the number of unoccupied (free) receptors and O1 and O2 represent the numbers of receptor-ligand complexes (or occupied receptors) attained by the respective pharmacological compounds. Rearranging for the total amount of receptor-ligand complexes, we have:

3 $${\rm{O}}1+{\rm{O}}2={{\rm{E}}}_{\max }-{\rm{UR}}$$

In a competition model, the binding of one ligand influences the binding of the other ligand. The ratios of the concentrations of the ligands and their respective binding affinities, in this case the respective EC_50_ values, determines the equilibrium. The following equations denote the allocations of the receptors to the different states:

4 $${\rm{O}}1=({\rm{C}}1/{{\rm{EC}}}_{50}1)\,{\rm{x}}\,{\rm{UR}}$$

5 $${\rm{O}}2=({\rm{C}}2/{{\rm{EC}}}_{50}2)\,{\rm{x}}\,{\rm{UR}}$$

where C1 and C2 are the plasma concentrations of two antipsychotic drugs, and EC_50_1 and EC_50_2 are the corresponding plasma concentrations for 50% receptor occupancies, as derived from published PET studies. By substituting these expressions in equation [2], we calculate

6 $${{\rm{E}}}_{\max }={\rm{UR}}\,{\rm{x}}(1+{\rm{C}}1/{{\rm{EC}}}_{50}1+{\rm{C}}2/{{\rm{EC}}}_{50}2)$$

To express the free receptor concentrations, we solve the equation for UR:

7 $${\rm{UR}}={{\rm{E}}}_{\max }/(1+{\rm{C}}1/{{\rm{EC}}}_{50}1+{\rm{C}}2/{{\rm{EC}}}_{50}2)$$

Inserting UR into equation [3], we obtain:

8 $${\rm{O}}1+{\rm{O}}2={{\rm{E}}}_{\max }-({{\rm{E}}}_{\max }/(1+{\rm{C}}1/{{\rm{EC}}}_{50}1+{\rm{C}}2/{{\rm{EC}}}_{50}2))$$

Since the receptor occupancy is a percentage of the maximum attainable receptor occupancy, i.e., 100%, we arrive at:

9 $${\rm{occupancy}}\,[ \% ]=100-(100/(1+{\rm{C}}1/{{\rm{EC}}}_{50}1+{\rm{C}}2/{{\rm{EC}}}_{50}2))$$

**Fig. 1:**
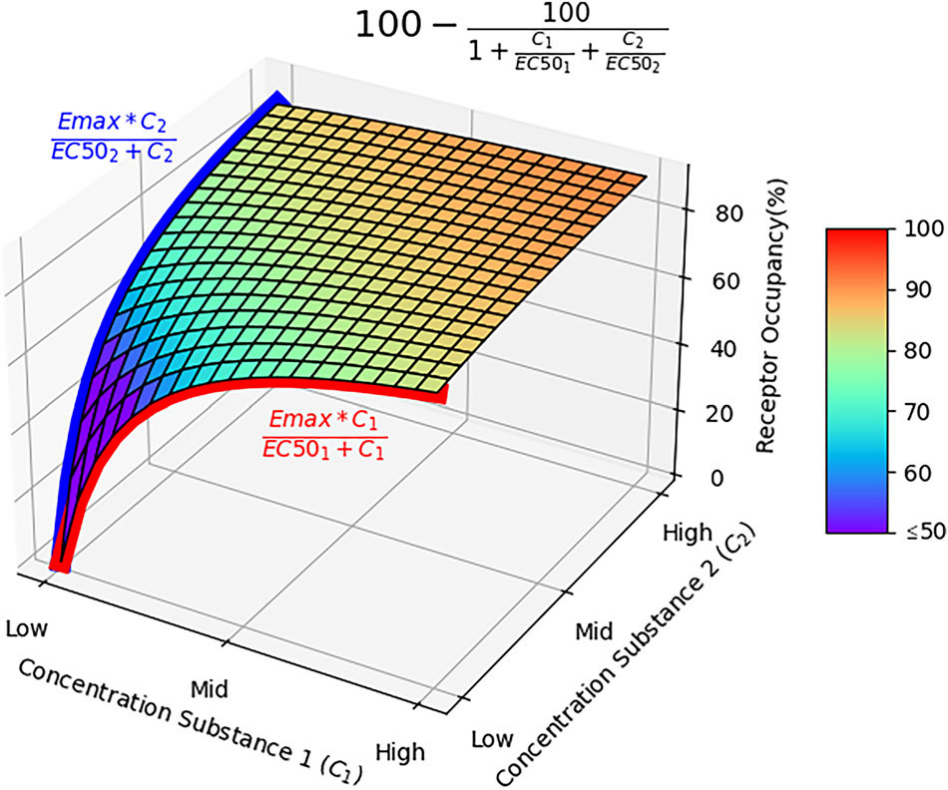
This figure illustrates the Michaelis-Menten diagram for two individual compounds with different EC50 and indicates the potential receptor occupancy when both compounds are combined. C1 = drug 1 serum concentration; C2 = drug serum concentration; EC_50_1 = drug 1 concentration for 50% receptor occupancy; EC_50_2 = drug 2 concentration for 50% receptor occupancy; E_max_ = maximum attainable receptor occupancy.

This extension of the Michaelis-Menten equation considers the competition between two different compounds with different affinities (Fig. 1). We undertook this analysis for binary combinations of five commonly used antipsychotics (aripiprazole, clozapine, haloperidol, olanzapine, risperidone), deriving their respective EC_50_ values from published PET studies. Our methodology however is also applicable for other dopaminergic drugs and could be extended to the case of treatments with more than two antipsychotics. We performed the mathematical and statistical analyses and generated figures using R Studio. The data and code used in this study are available upon request by contacting the authors.

### EC_50_ values

We derived EC_50_ values for the five antipsychotic medications from an exhaustive search of published PET and SPECT studies reporting D_2_R occupancy as a function of plasma drug concentrations. For three of the compounds (haloperidol, olanzapine, risperidone), the calculations are based on the EC_50_ values reported by Uchida et al. [32]. Our systematic search of the literature did not yield further reports of EC_50_ for olanzapine, clozapine, and risperidone [28]. There was one study giving an independent estimate of the EC_50_ for haloperidol [36], closely matching in magnitude the report of Uchida et al. [32]. Uchida and his colleagues recommended the general use of an unconstrained model, without fixing E_max_ at 100% [32]. Thus, we used the unconstrained model for describing the EC_50_ values of haloperidol, olanzapine and risperidone. The constrained model assumes that D_2_Rs are entirely (100%) occupiable by the antipsychotic. For most antipsychotics, E_max_ values derived with an unconstrained model are close to 100%, such that EC_50_ values estimated from the constrained and the unconstrained models do not substantially differ. For example, the EC_50_ estimated for haloperidol was 0.32 ng/mL for the unconstrained model and 0.70 ng/mL when E_max_ was constrained to 100%. For olanzapine, the respective values are 7 ng/mL and 10 ng/mL, and for risperidone 5 ng/mL and 8 ng/mL.

The pharmacokinetic modelling of clozapine is more intricate compared to the aforementioned compounds. Using the unconstrained model, Uchida et al. calculated a maximum attainable D_2_R occupancy of only 60%, with a respective EC_50_ of 105 ng/mL. The constrained model provided a substantially higher EC_50_ value of 483 ng/mL. However, clozapine could plausibly occupy more than 60% of striatal D_2_Rs in patients: high doses of clozapine occupied more than 80% of D_2_R in striatum of non-human primate [37]. Almost all PET studies that determined D_2_R occupancy by clozapine used [^11^C]raclopride as the radiotracer. In a [^18^F]fallypride PET study, using an unconstrained model, we found an E_max_ indicating nearly complete receptor saturation by clozapine, with EC_50_ values of 950 ng/mL for the putamen and 582 ng/mL for the caudate [38]. These values seem to be biologically and clinically plausible, since the therapeutic reference range for clozapine is 350 – 600 ng/mL, and even much higher plasma concentrations do not evoke extrapyramidal side effects. For a subset of patients, even higher clozapine plasma concentrations might be required to achieve optimal clinical response [39]. Therefore, we used the clozapine EC_50_ value from the constrained model provided by Uchida et al. Calculations with even higher EC50 values can be found in the supplement (Supplementary Tables 11–14).

There are no reports on D_2_R occupancy by clozapine since 2011.

For aripiprazole, there are seven PET studies in human subjects reporting D_2_R occupancy values [40–46], of which only two report EC_50_ values (or individual plasma concentrations for calculation of a population-based EC_50_). In one of the studies, Takahata et al. calculated an EC_50_ of 10 ng/mL for the whole striatum and 12 ng/mL for the putamen [45], versus the finding of Gründer et al. of 10 ng/mL for the putamen and 9 ng/mL for the caudate [40]. Therefore, we based our present occupancy calculations on an EC_50_ value of 10 ng/mL.

Aripiprazole’s main metabolite, dehydroaripiprazole, though, also occupies the D2 receptor, but only one study reports estimates for EC50 that are based on active moiety (aripiprazole + dehydroaripiprazole) concentrations of the drug ([40]: putamen 20 ng/mL, caudate 18 ng/mL). Calculations with the adjusted EC50 and therapeutic reference range can be found in the supplementary material. Based on the EC_50_ values presented in Table 1, we calculated the expected D_2_R receptor occupancies under combination treatment with two different antipsychotics.

**Table 1:** The table shows the 50% “effective concentration” (EC_50_) concentration associated with half-maximal occupancy of the respective antipsychotic substances (ng/ml = nanogram per milliliter).

Substance	Aripiprazole	Clozapine	Haloperidol	Olanzapine	Risperidone
**EC**_**50**_ **value (ng/ml)**	**10**	**483**	**0.32**	**7**	**5**

### Calculation of D_2/3_ receptor occupancy under combination treatment

The five antipsychotic drugs under consideration give ten different binary combinations, for which we calculated the D_2_R occupancies according to Eq. 9. We modelled the occupancies for wide a range of plasma drug concentrations extending well above and below the therapeutic reference range [47].

## Results

**Fig. 2:**
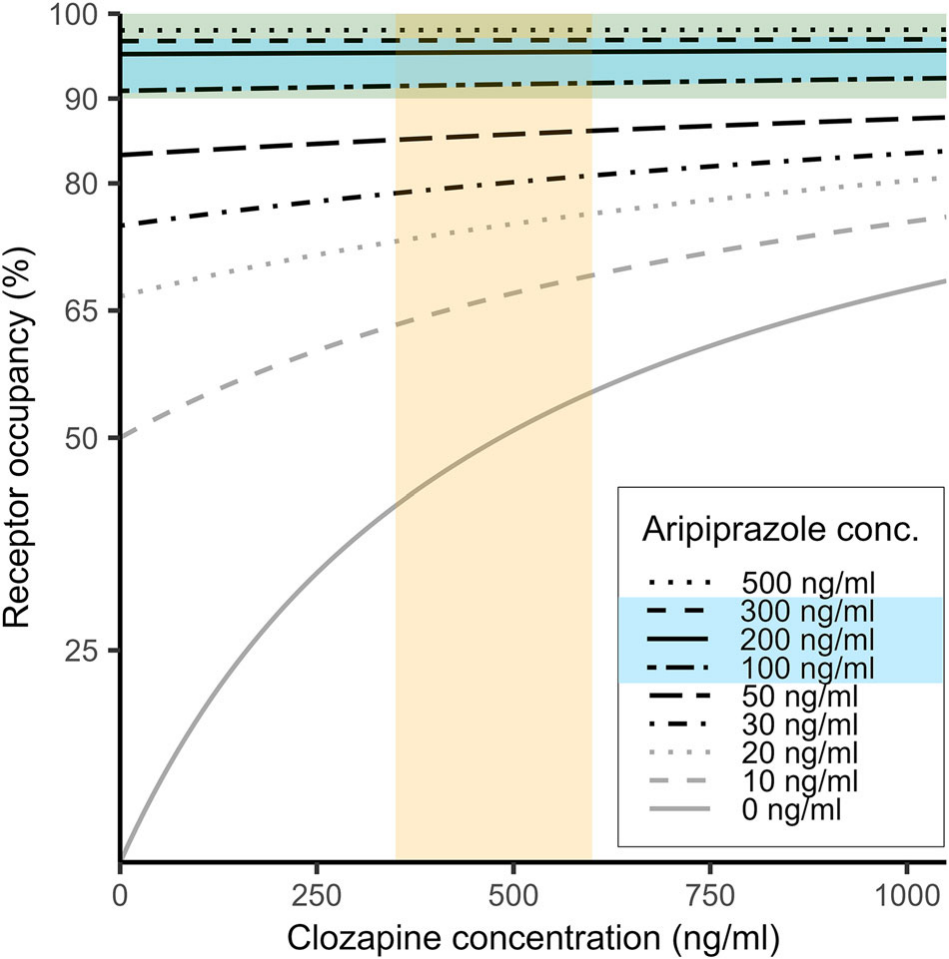
Model curves illustrating total dopamine D_2/3_ receptor occupancy in striatum under treatment with clozapine at increasing plasma concentrations of aripiprazole (range 0 – 500 ng/ml). The yellow rectangular field indicates the therapeutic reference range of the plasma clozapine concentration; the blue field indicates the therapeutic reference range of the plasma aripiprazole concentration; the green rectangular field indicates the “therapeutic window” of receptor occupancy for clozapine & aripiprazole. conc. = concentration, ng/ml = nanogram per milliliter.

We present calculated D_2_R occupancies under treatment with the ten binary combinations of clinically common antipsychotic medications over broad ranges of plasma concentrations in Figs. 2 and 4 and Supplementary Figures 1–7 (see Supplementary Information). Supplementary Tables 1–10 show the exact values presented in the Supplementary Information. We highlight three representative forms of drug combinations, i.e. clozapine augmentation with aripiprazole, clozapine augmentation with haloperidol and olanzapine combination with risperidone. These combinations arise frequently in the clinical setting, but gave distinctly different net occupancies according to our model.

### Clozapine augmentation with aripiprazole

Clozapine augmentation with the partial agonist aripiprazole is the most effective antipsychotic combination [13], and is the first recommended strategy according to the available guidelines when clozapine monotherapy has not led to a sufficient response [48]. Disregarding its active metabolite norclozapine, the therapeutic reference range for the plasma clozapine level is generally 350–600 ng/ml, although the upper limit is still debated [39, 49]. The augmentation of clozapine with aripiprazole favorably influenced negative symptoms in meta-analysis [9], and led to the fewest re-hospitalizations among various augmentations in a nationwide cohort study [13].

Despite their common action at striatal D_2_R, neither aripiprazole nor clozapine have a strict upper limit of therapeutic 80% occupancy, which contributes to their favorable side-effect profile. Given EC_50_ values of 10 ng/mL for aripiprazole and 483 ng/mL for clozapine, our model predicts that combination treatment with these drugs at plasma concentrations at the low ends of the respective recommended therapeutic reference ranges (aripiprazole: 100 ng/mL; clozapine 400 ng/mL) will lead to 92% D_2_R occupancy (Fig. 2). Monotherapy with aripiprazole at 100 ng/mL plasma concentration results in negligibly lower occupancy (91%). An additional plasma clozapine concentration of 1000 ng/mL, considerably exceeding the recommended monotherapy levels, would increase striatal D_2_R occupancy by just 0.5%. With aripiprazole plasma concentrations exceeding 100 ng/mL, addition of clozapine would only negligibly increase occupancy.

Given the therapeutic range of 100–350 ng/ml for aripiprazole plasma levels, and the requirement for therapeutic occupancy exceeding 90% for the partial agonist [20, 31], our modeling shows that the reference ranges of clozapine and aripiprazole monotherapies would likewise serve for their combined administration. This might partly explain the favorable clinical risk-benefit ratio with the clozapine/aripiprazole combination.

### Clozapine augmentation with haloperidol

Combination of clozapine with haloperidol is especially relevant for our model, because clinical PET occupancy data are available for this combination [34], and no other binary APP. The therapeutic reference range for haloperidol plasma concentrations is 1 – 10 ng/ml [47]. A randomized clinical trial showed comparable antipsychotic efficacy, but more pronounced extrapyramidal symptoms, for clozapine augmentation with haloperidol as compared to clozapine augmentation with aripiprazole [50].

**Fig. 3:**
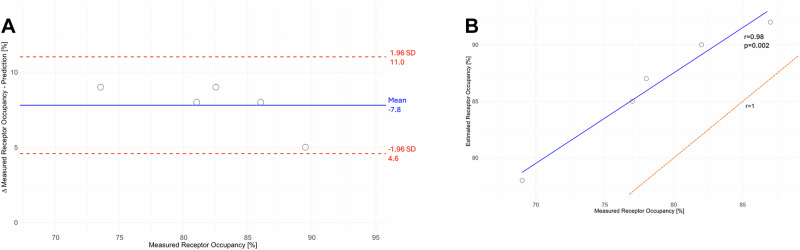
Agreement between model-predicted and measured D_2/3_ receptor occupancy. **A** Bland-Altman plot showing the agreement between model-predicted and actual shared receptor occupancy for the clozapine-haloperidol combination from Kapur et al. [34]. The mean absolute difference is 7.8 ± 1.6% (range 5–9%). Individual differences are represented as points, the mean difference as a blue line, and the standard deviation in both directions as red lines. **B** Scatter plot showing the correlation between measured receptor occupancy (from Kapur et al. [34]) and estimated D_2_R occupancy (from our model). Blue line - correlation between estimated and measured receptor occupancy; Orange dotted line - line of unity: represents the hypothetical ideal 1:1 relationship where predicted values perfectly match the measured values.

Our model predicts a mean ± standard deviation (SD) absolute difference of 7.8 ± 1.6% (range 5 – 9%) between the estimated D_2_R occupancy at the indicated plasma concentrations and the receptor occupancy measured by PET (Fig. 3A) [32]. There was a high correlation between the observed and predicted values of D_2_R occupancy (*r* = 0.98, *p* = 0.002; Fig. 3B). All predicted values are higher than the measured data, suggesting that our model exhibits a tendency to slightly overestimate receptor occupancy in both figures (see comparison with the ‘Line of Unity’, Fig. 3B). The magnitude of the mean (SD) absolute difference closely matches those reported for the individual substances, namely 12.1 ± 9.1% for clozapine and 7.0 ± 7.1% for haloperidol [30] (further information in the supplementary information).

**Fig. 4:**
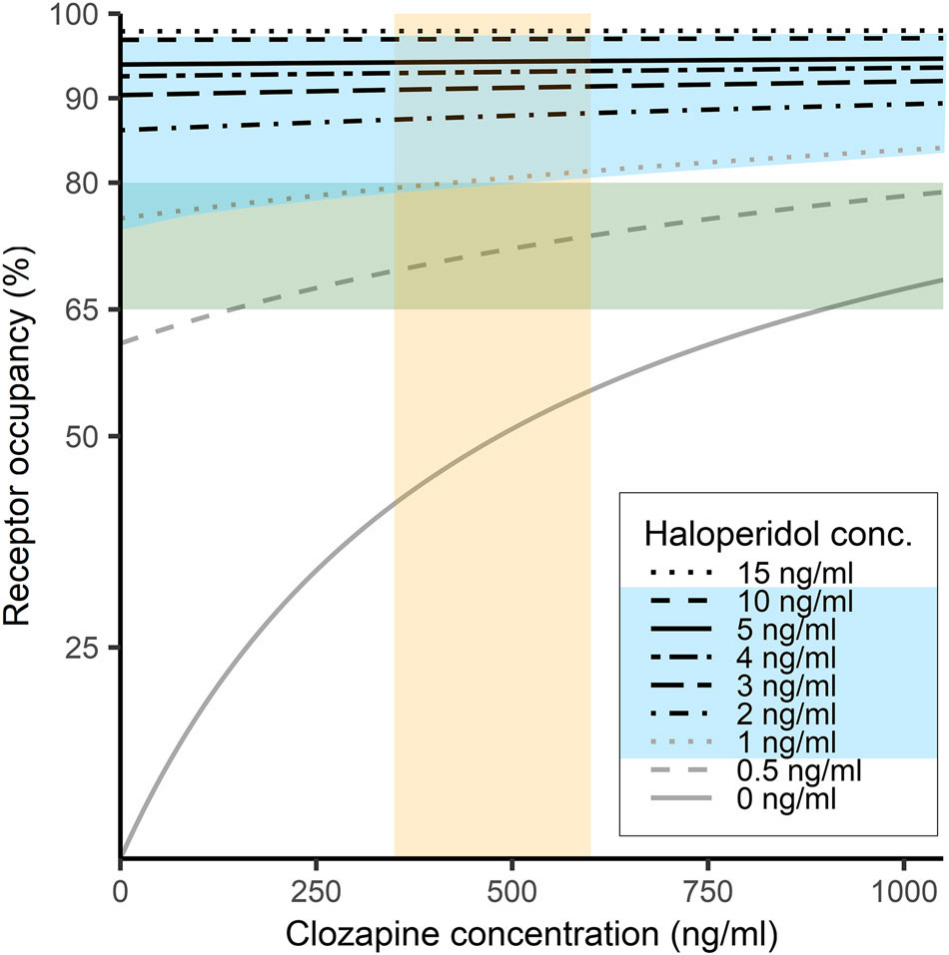
Model curves illustrating total dopamine D_2/3_ receptor occupancy in striatum under treatment with clozapine at increasing plasma concentrations of haloperidol (range 0 – 15 ng/ml). The yellow rectangular field indicates the therapeutic reference range of the plasma clozapine concentration; the blue field indicates the therapeutic reference range of the plasma haloperidol concentration; the green rectangular field indicates the “therapeutic window” of receptor occupancy for clozapine & haloperidol. conc. = concentration, ng/ml = nanogram per milliliter.

In accord with the available clinical data [50], our model predicts the attainment of an effective receptor net occupancy even with low doses. The modelling also suggests that net occupancy with haloperidol augmentation could more easily exceed the recommended limit range as compared to clozapine augmentation with aripiprazole (Fig. 4).

### Two D_2/3_ receptor antagonists

#### Combination of olanzapine with risperidone

The combination of olanzapine with risperidone is an example with frequent application in clinical practice of APP with two drugs having similar affinities for D_2_Rs. In contrast to clozapine, the antipsychotic efficacy and associated psychomotor side effects of olanzapine bear a strong relation with striatal D_2_R occupancy [28]. While an expert consensus proposes a reference plasma olanzapine concentration in the range of 20 to 80 ng/ml for oral and long acting injectable (LAI) formulations [47], authors of a recent systematic meta-analysis suggested a narrower therapeutic window of 20 to 40 ng/ml [51].

For risperidone, Nyberg et al. observed the target 65–80% occupancy of D_2_Rs with a plasma concentration range for the active moiety (sum of risperidone and its active metabolite) in the range of 13 – 28 ng/ml [52]. Nearly all patients exceeding the 80% occupancy threshold developed extrapyramidal symptoms [52]. For the LAI, a lower threshold of around 50% has been proposed [53].

**Fig. 5:**
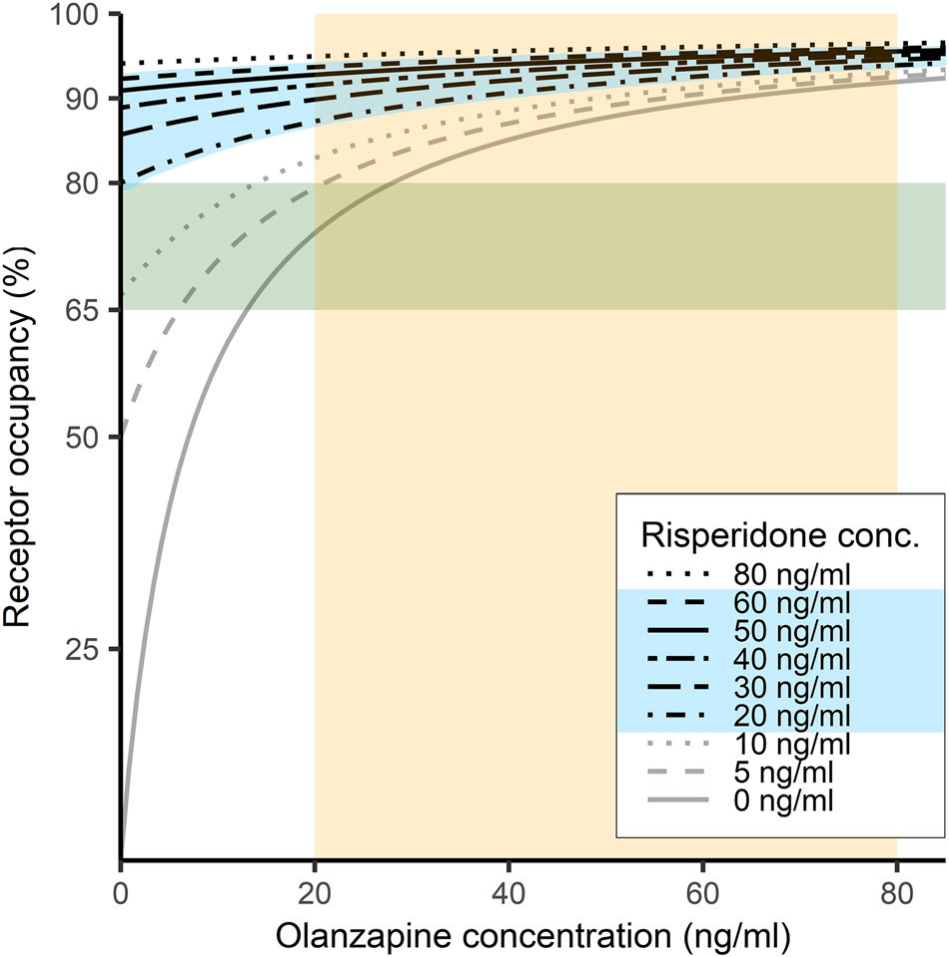
Model curves illustrating total dopamine D_2/3_ receptor occupancy in striatum under treatment with olanzapine at increasing plasma concentrations of risperidone (range 0 – 80 ng/ml). The yellow rectangular field indicates the therapeutic reference range of the plasma olanzapine concentration; the blue field indicates the therapeutic reference range of the plasma risperidone concentration; the green rectangular field indicates the “therapeutic window” of receptor occupancy for olanzapine & risperidone. conc. = concentration, ng/ml = nanogram per milliliter.

Augmentation of olanzapine monotherapy often entails risperidone in an LAI formulation [13]. In monotherapies, plasma concentrations of 10 ng/ml, which are below their respective recommended reference ranges [47], lead to striatal D_2_R occupancies of 59% for olanzapine and 67% for risperidone active moiety (together with its active metabolite paliperidone/9-hydroxyrisperidone). We calculate in our model that APP with low doses of both compounds would lead to 77% net occupancy at striatal D_2_Rs, which falls just below the 80% threshold for antagonists that is generally associated with a high risk of extrapyramidal side effects (Fig. 5).

As with other APP combinations with high affinities for D_2_Rs, our pharmacokinetic calculations show that rather low plasma concentrations of olanzapine/risperidone should obtain high occupancy. The model predicts “safe windows” in the ranges of 10 - 30 ng/ml olanzapine and 5 - 10 ng/ml risperidone. We also see that higher plasma concentrations obtain a “point of futility” or saturation effect regarding net antagonism at D_2_Rs [54]. Thus, PET-derived occupancy estimates predict a narrow therapeutic window for the olanzapine/risperidone combination therapy.

## Discussion

We developed a law of mass action-derived modelling approach for calculating receptor occupancy during APP with clinically relevant medication pairs. Results of this novel model illustrate that (without dose adjustment) combination of two compounds with moderate to high receptor affinity for striatal D_2_Rs should readily attain a net occupancy in excess of the 80% threshold level for extrapyramidal side effects. Consequently, when using antipsychotic polypharmacy, reference ranges should be lower. Augmentation with the partial agonist aripiprazole might be an exception, as demonstrated for the present case with clozapine. The clinical relevance of our model derives from its potential to guide therapeutic drug monitoring in the context of antipsychotic polypharmacy. When clinicians consider antipsychotic augmentation, our model could guide the adjustment of target ranges of the plasma drug concentrations. Furthermore, our approach could yield new therapeutic reference ranges specifically for APP, thereby improving the efficacy and safety of polypharmacology.

We note inherent limitations of our modelling approach in relation to its theoretical nature. First, we must concede the possibility of occupancy bias in the monotherapy PET studies that form the basis of our model. While striatal D_2_R availability in the unblocked state is usually measured in healthy subjects, corresponding studies in a blocked condition, with few exceptions, are usually determined in patients under treatment with the compound. However, endogenous dopamine concentrations and D_2_R density differ in some patients compared to healthy subjects [55, 56], and may be perturbed due to effects of treatment.

Furthermore, PET studies of D_2_R occupancy seldom account for competition from endogenous dopamine, which typically increases upon blockade of presynaptic autoreceptors. While this may have limited impact on high-affinity compounds, it could be significant for antipsychotics like clozapine or quetiapine [38, 57, 58]. However, present methods do not enable incorporation of perturbed competition from endogenous dopamine into an interpretation of the EC50 value in PET studies, either in antipsychotic monotherapy, or in combination therapy.

Moreover, our model does not accommodate confounders potentially arising in real world scenarios, such as inter-individual differences in striatal D_2_R density, ligand affinity, or drug partitioning between blood and brain. We note that pharmacokinetic variability in drug absorption, plasma protein binding, metabolism or elimination, and drug-drug interactions in general, do not influence our model estimates, which are robust to individual variability in plasma concentrations of antipsychotics. Put another way, the model describes the relationship between plasma concentrations and net receptor occupancy, irrespective of any pharmacokinetic factors that may have influenced plasma concentrations. The situation is theoretically somewhat different for the case of blood-brain-barrier permeability but even here, the partitioning between blood and brain is assessed by the experimentally determined EC50. For example, a compound with poor brain permeability (e.g., amisulpride) will have a relatively high EC50 as determined in PET studies, despite having a high D_2_R binding affinity in vitro [59]. Thus, while individual heterogeneity of D_2_R receptor availability in patients versus controls and genetic variability of transporters of the blood-brain barrier (e.g. ABCB1 transporters) could contribute to inaccuracy of our model, we do not expect that pharmacokinetic factors would influence model-based occupancy estimation.

Furthermore, Uchida et al. demonstrated that a simplified binding model served to estimate receptor occupancy with monotherapy [32], and our extended model accurately predicted occupancy for the only APP combination documented by PET (i.e. haloperidol augmentation of clozapine [34]). The slight overestimation in our model compared to the measured occupancy values could arise from our selection of EC50 values (in this case of haloperidol) based on application of an unconstrained model to the PET data.

In addition, pharmacodynamic drug-drug interactions could potentially influence receptor occupancy. This might be especially the case when combining a D_2_R antagonist with aripiprazole or other partial agonists. Augmentation with partial agonists, due to their inherently high affinity for D_2_Rs, tend to counteract the extrapyramidal side effects associated with D_2_R antagonists by competitive binding at their common receptor target (e.g. [60]). In APP, the antipsychotic agent with the higher affinity typically displaces the compound with weaker affinity [4]. This scenario led to the suggestion that partial agonists like aripiprazole should generally not have dose limitations in APP, due to their intrinsic D_2_R agonism even at complete occupancy [61]. On the other hand, D_2_R antagonists may somewhat counteract the therapeutic benefits mediated by partial agonists like aripiprazole or cariprazine, although the partial agonists have the highest D_2_ affinities of all antipsychotics [20]. While our model provides a pharmacodynamic rationale for Reynolds’ proposition regarding the combination of clozapine (or quetiapine, i.e., a drug with even lower D_2_R affinity) with a partial agonist like aripiprazole, it does not provide the same rationale for aripiprazole augmentation of antipsychotics other than clozapine or quetiapine. There is likely some critical concentration threshold where aripiprazole levels largely displace the antagonist compound, thereby shifting the “therapeutic window” of 60–80% D_2_R occupancy. By definition, a partial agonist exerts a moderate receptor activation (intrinsic activity < 1), which should suffice to avoid EPS, even with complete occupancy [62]. However, our present model cannot determine when this threshold would be reached or how low the concentration of the antagonist compound must be, which represents a limitation of our model.

Despite these limitations, our finding that striatal D_2_R occupancy would readily surpass the 65–80% threshold at low to moderate plasma concentrations held for all ten of the binary combinations. We focused on APP with the five antipsychotics that count among the most used in clinical practice, and the most frequently employed in binary APP combinations [13].

While the targeting of multiple neuroreceptors provides a rationale for APP [7], multiple antipsychotics will have an additive effect on D_2_R occupancy. The combination of olanzapine with risperidone, among the most common instances in the clinic [13], presents a case in point; [^11^C]doxepine PET confirmed that olanzapine occupies histamine H_1_ receptors in human brain [63], which doubtlessly imparts additional sedation for the patient. However, our analysis shows that combining the two substances could easily result in excessive net blockade of striatal D_2_Rs, resulting in EPS and other deleterious side effects.

Our model analyses have clear implications for antipsychotic polypharmacy and for the treatment of schizophrenia in general. A study conducted in 2011, involving 127 patients with schizophrenia who had achieved stability with APP, revealed that when these patients were randomly assigned to either continue with APP or switch to antipsychotic monotherapy, 86% of those who remained on APP continued with both medications, whereas 69% of those who switched to monotherapy were able to tolerate and continue with it [64]. It has been suggested that this implies that more patients may be receiving APP treatment than required [65]. Applying our results to this logic, it could be argued that not only do 69% of patients on APP not receive additional benefits from a second antipsychotic, but they are also at a high risk of overloading their D_2_Rs.

This is especially relevant in the context of potential long-term side effects of excessive D_2_R antagonism, notably tardive dyskinesia [66] and super-sensitivity psychosis [67–69]. Moreover, we argue that additive D_2_R antagonism cannot account for the benefits of APP as compared to monotherapy. The case is stronger for drug augmentation that brings additional pharmacological effects, such as histamine H_1_ receptor blockade or 5HT_2A_ antagonism.

In summary, we present an extended Michaelis-Menten mass action model for estimating net occupancy at striatal D_2_Rs during polypharmacy with antipsychotic medications. Model results indicate that binary combinations among five widely used antipsychotics are apt to exceed the recommended 80% antagonist occupancy unless there is substantial and substance-specific adjustment of doses, thereby reducing plasma levels.

Recommendations for enabling dose adjustment in antipsychotic combination therapy have already been outlined. For example, the more commonly employed ‘percentage method’ entails calculating the percentage of the maximum licensed dose for each co-prescribed anti-psychotic medication. A dose summation exceeding 100% is classified as a high dose [70]. However, this simple approach does not support either pharmacokinetic or pharmacodynamic adjustments regarding the individual dose combinations. Furthermore, for many combinations, the existing recommendations would still result in net antagonist occupancy exceeding the recommended 80%. Therapeutic drug monitoring is already known to be an evidence-based method for optimizing and personalizing monotherapy with antipsychotics [54, 71, 72]. Application of our occupancy model could be a first step towards establishing therapeutic reference ranges for various antipsychotic combinations. Ultimately, a full validation of our model calls for empirical testing, through follow-up PET studies to measure the net D_2_R occupancy achieved with different combination therapies (including combinations with partial agonists) in patients with schizophrenia.

## Supplementary information

Supplemental Material

## Data Availability

All data and code are available from the authors upon reasonable request.
