# Supplementary material for: The sense and nonsense of antipsychotic combinations: A model for dopamine D2/3 receptor occupancy
Source: Transl Psychiatry. 2025 Oct 1;15:348. doi: 10.1038/s41398-025-03582-2 (PMC12488982; doi:10.1038/s41398-025-03582-2)
Supplement: Supplementary file 1 — Supplemental Material [file 41398_2025_3582_MOESM1_ESM.docx]

**SUPPLEMENTARY INFORMATION (SI)**

1. **Supplementary Figures**


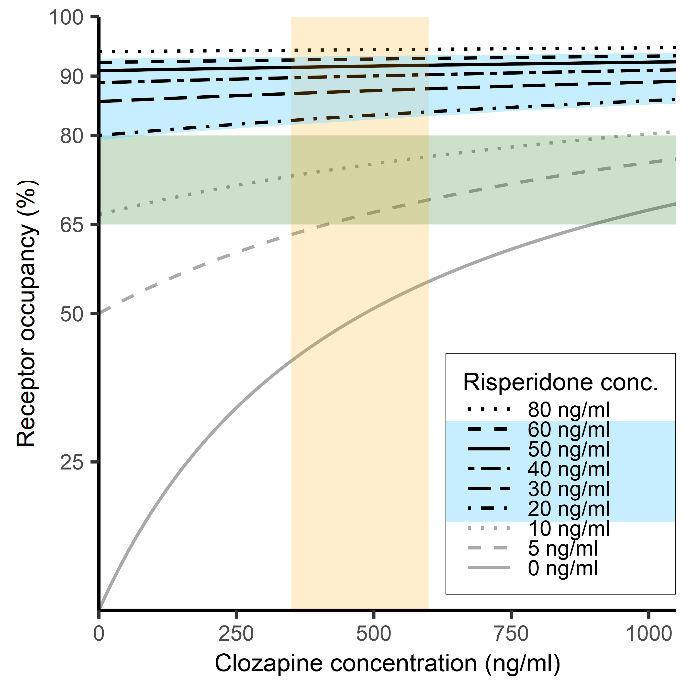


*Supplementary Figure 1: Model curves illustrating total dopamine D2/3 receptor occupancy in striatum under treatment with clozapine at increasing plasma concentrations of risperidone (range 0 – 80 ng/ml). The yellow rectangular field indicates the therapeutic reference range of the plasma clozapine concentration; the blue field indicates the therapeutic reference range of the plasma risperidone concentration; the green rectangular field indicates the “therapeutic window” of receptor occupancy for clozapine & risperidone. conc. = concentration, ng/ml = nanogram per milliliter.*


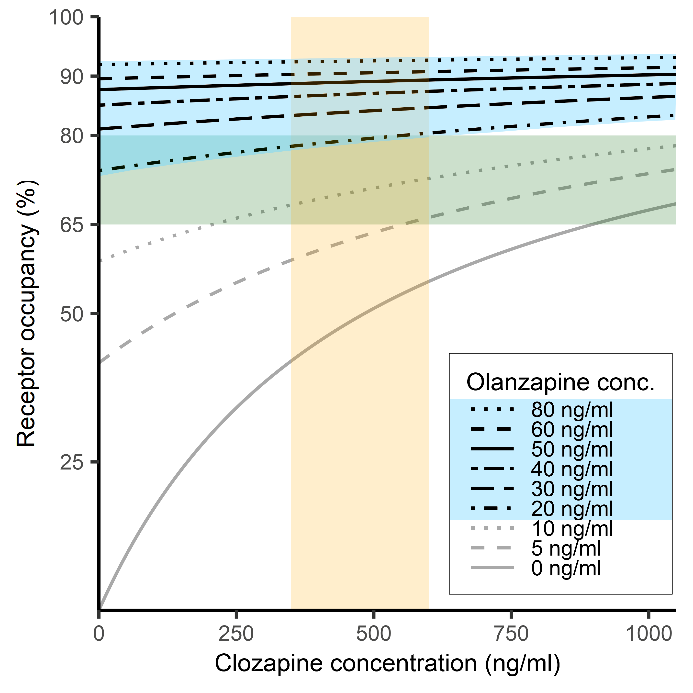


*Supplementary Figure 2:* *Model curves illustrating total dopamine D2/3 receptor occupancy in striatum under treatment with clozapine at increasing plasma concentrations of olanzapine (range 0 – 80 ng/ml). The yellow rectangular field indicates the therapeutic reference range of the plasma clozapine concentration; the blue field indicates the therapeutic reference range of the plasma olanzapine concentration; the green rectangular field indicates the “therapeutic window” of receptor occupancy for clozapine & olanzapine. conc. = concentration, ng/ml = nanogram per milliliter.*

*
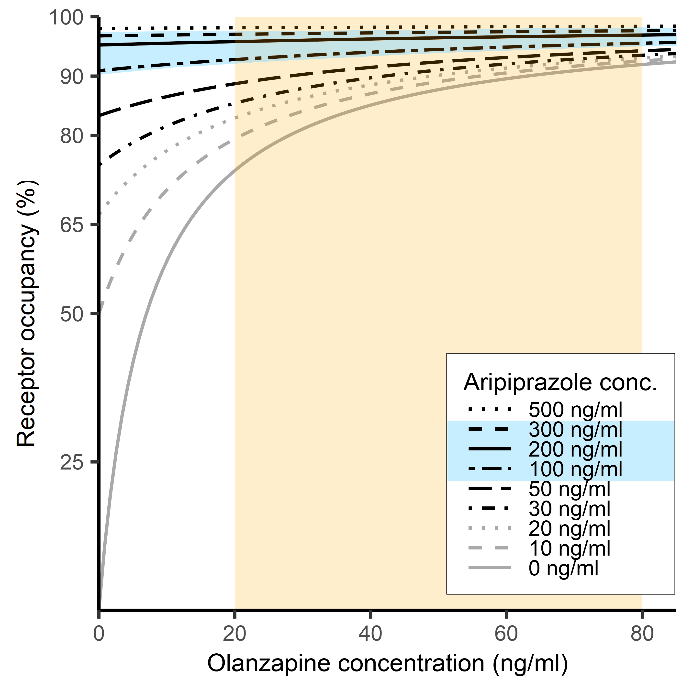
*

*Supplementary Figure 3: Model curves illustrating total dopamine D2/3 receptor occupancy in striatum under treatment with olanzapine at increasing plasma concentrations of aripiprazole (range 0 – 500 ng/ml). The yellow rectangular field indicates the therapeutic reference range of the plasma olanzapine concentration; the blue field indicates the therapeutic reference range of the plasma aripiprazole concentration. conc. = concentration, ng/ml = nanogram per milliliter.*


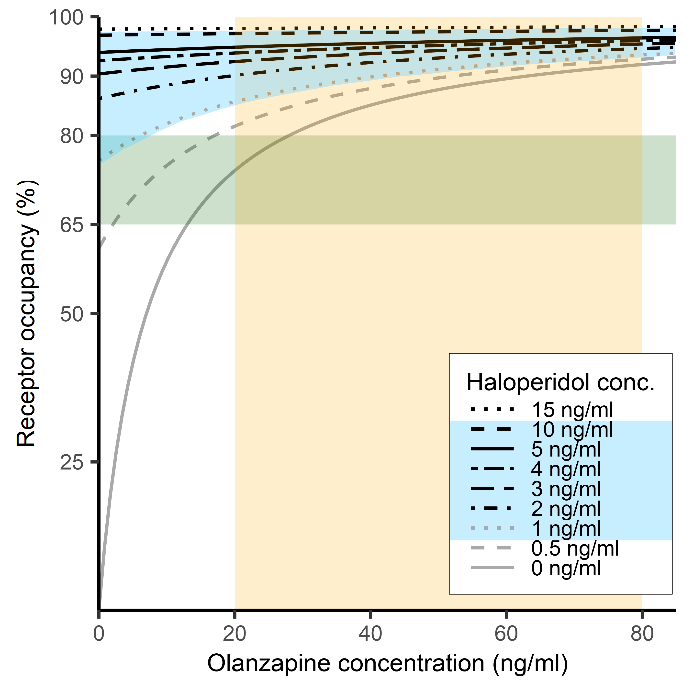


*Supplementary Figure 4:* *Model curves illustrating total dopamine D2/3 receptor occupancy in striatum under treatment with olanzapine at increasing plasma concentrations of haloperidol (range 0 – 15 ng/ml). The yellow rectangular field indicates the therapeutic reference range of the plasma olanzapine concentration; the blue field indicates the therapeutic reference range of the plasma aripiprazole concentration; the green rectangular field indicates the “therapeutic window” of receptor occupancy for olanzapine & haloperidol. conc. = concentration, ng/ml = nanogram per milliliter.*

*
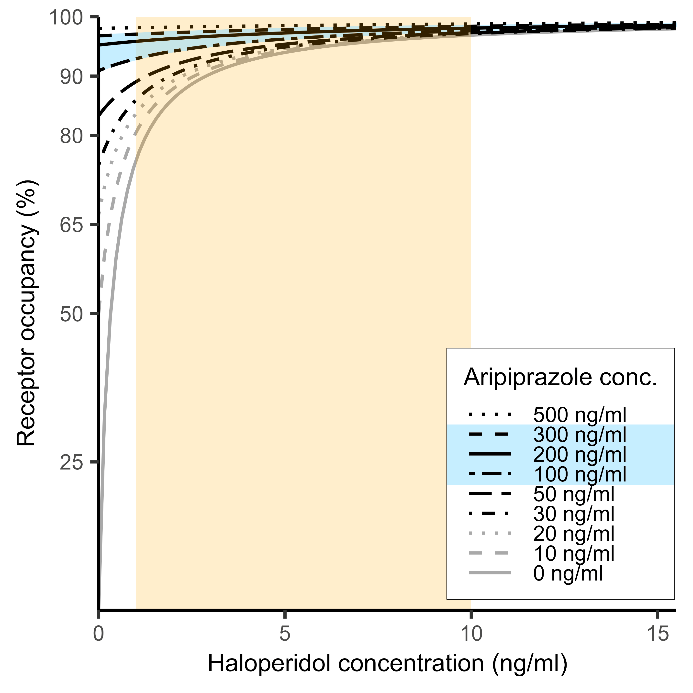
*

*Supplementary Figure 5: Model curves illustrating total dopamine D2/3 receptor occupancy in striatum under treatment with haloperidol at increasing plasma concentrations of aripiprazole (range 0 – 500 ng/ml). The yellow rectangular field indicates the therapeutic reference range of the plasma haloperidol concentration; the blue field indicates the therapeutic reference range of the plasma aripiprazole concentration conc. = concentration, ng/ml = nanogram per milliliter.*

*
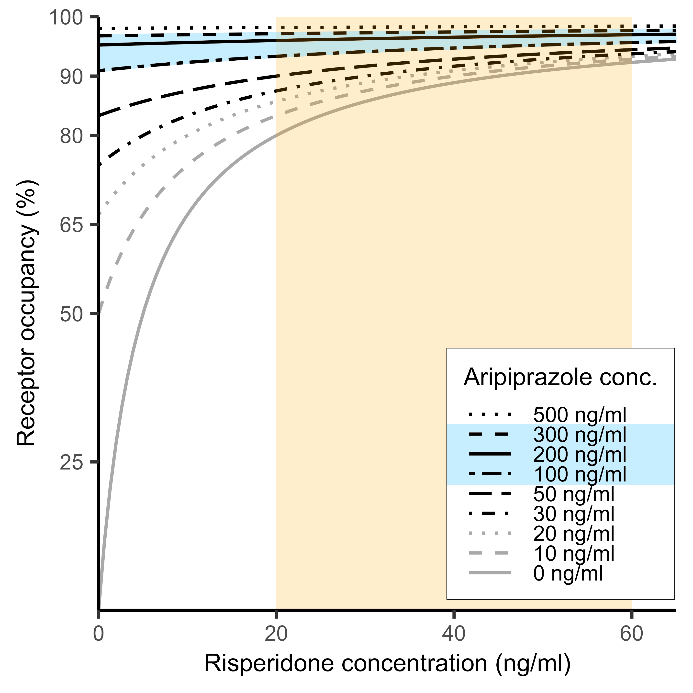
*

*Supplementary Figure 6: Model curves illustrating total dopamine D2/3 receptor occupancy in striatum under treatment with risperidone at increasing plasma concentrations of aripiprazole (range 0 – 500 ng/ml). The yellow rectangular field indicates the therapeutic reference range of the plasma risperidone concentration; the blue field indicates the therapeutic reference range of the plasma aripiprazole concentration. conc. = concentration, ng/ml = nanogram per milliliter.*


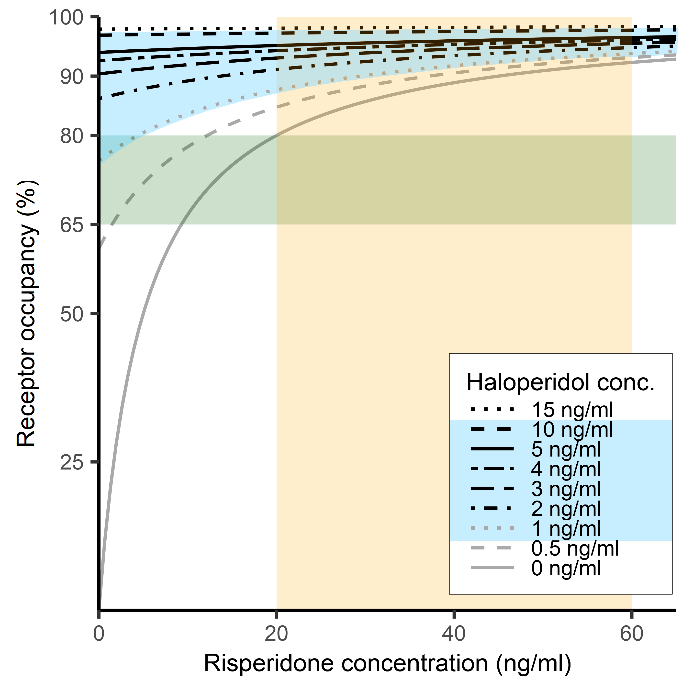


*Supplementary Figure 7:* *Model curves illustrating total dopamine D2/3 receptor occupancy in striatum under treatment with risperidone at increasing plasma concentrations of haloperidol (range 0 – 15 ng/ml). The yellow rectangular field indicates the therapeutic reference range of the plasma risperidone concentration; the blue field indicates the therapeutic reference range of the plasma aripiprazole concentration; the green rectangular field indicates the “therapeutic window” of receptor occupancy for risperidone & haloperidol. conc. = concentration, ng/ml = nanogram per milliliter.*

1. **Supplementary Tables**

*Supplementary Table 1*

| **Plasma Concentration [ng/mL]** | | **Haloperidol** | | | | | | |
| --- | --- | --- | --- | --- | --- | --- | --- | --- |
|  |  | **0** | **1** | **2** | **3** | **5** | **10** | **15** |
| **Clozapine** | **0** | **0** | **76** | **86** | **90** | **94** | **97** | **98** |
|  | **200** | **29** | **78** | **87** | **91** | **94** | **97** | **98** |
|  | **400** | **45** | **80** | **88** | **91** | **94** | **97** | **98** |
|  | **600** | **55** | **81** | **88** | **91** | **94** | **97** | **98** |
|  | **800** | **62** | **83** | **89** | **92** | **95** | **97** | **98** |
|  | **1000** | **67** | **84** | **89** | **92** | **95** | **97** | **98** |
|  | **1200** | **71** | **85** | **90** | **92** | **95** | **97** | **98** |

*Supplementary Table 2*

| **Plasma Concentration [ng/ml]** | | **Aripiprazole** | | | | | | |
| --- | --- | --- | --- | --- | --- | --- | --- | --- |
|  |  | **0** | **50** | **100** | **200** | **300** | **500** | **1000** |
| **Clozapine** | **0** | **0** | **83** | **91** | **95** | **97** | **98** | **99** |
|  | **200** | **29** | **84** | **91** | **95** | **97** | **98** | **99** |
|  | **400** | **45** | **85** | **92** | **95** | **97** | **98** | **99** |
|  | **600** | **55** | **86** | **92** | **96** | **97** | **98** | **99** |
|  | **800** | **62** | **87** | **92** | **96** | **97** | **98** | **99** |
|  | **1000** | **67** | **88** | **92** | **96** | **97** | **98** | **99** |
|  | **1200** | **71** | **88** | **93** | **96** | **97** | **98** | **99** |

*Supplementary Table 3*

| **Plasma Concentration [ng/mL]** | | **Olanzapine** | | | | | | |
| --- | --- | --- | --- | --- | --- | --- | --- | --- |
|  |  | **0** | **10** | **20** | **30** | **40** | **60** | **100** |
| **Clozapine** | **0** | **0** | **59** | **74** | **81** | **85** | **90** | **93** |
|  | **200** | **29** | **65** | **77** | **82** | **86** | **90** | **94** |
|  | **400** | **45** | **69** | **79** | **84** | **87** | **90** | **94** |
|  | **600** | **55** | **73** | **80** | **85** | **87** | **91** | **94** |
|  | **800** | **62** | **76** | **82** | **86** | **88** | **91** | **94** |
|  | **1000** | **67** | **78** | **83** | **86** | **89** | **91** | **94** |
|  | **1200** | **71** | **80** | **84** | **87** | **89** | **92** | **94** |

*Supplementary Table 4*

| **Plasma**  **Concentration [ng/mL]** | | **Risperidone** | | | | | | |
| --- | --- | --- | --- | --- | --- | --- | --- | --- |
|  |  | **0** | **10** | **20** | **30** | **40** | **60** | **100** |
| **Clozapine** | **0** | **0** | **67** | **80** | **86** | **89** | **92** | **95** |
|  | **200** | **29** | **71** | **82** | **87** | **89** | **93** | **95** |
|  | **400** | **45** | **74** | **83** | **87** | **90** | **93** | **95** |
|  | **600** | **55** | **76** | **84** | **88** | **90** | **93** | **96** |
|  | **800** | **62** | **79** | **85** | **88** | **91** | **93** | **96** |
|  | **1000** | **67** | **80** | **86** | **89** | **91** | **93** | **96** |
|  | **1200** | **71** | **82** | **87** | **89** | **91** | **94** | **96** |

*Supplementary Table 5*

| **Plasma Concentration [ng/ml]** | | **Risperidone** | | | | | | |
| --- | --- | --- | --- | --- | --- | --- | --- | --- |
|  |  | **0** | **10** | **20** | **30** | **40** | **60** | **100** |
| **Olanzapine** | **0** | **0** | **67** | **80** | **86** | **89** | **92** | **95** |
|  | **10** | **59** | **77** | **84** | **88** | **90** | **93** | **96** |
|  | **20** | **74** | **83** | **87** | **90** | **92** | **94** | **96** |
|  | **30** | **81** | **86** | **89** | **91** | **92** | **94** | **96** |
|  | **40** | **85** | **89** | **91** | **92** | **93** | **95** | **96** |
|  | **60** | **90** | **91** | **93** | **94** | **94** | **95** | **97** |
|  | **100** | **93** | **94** | **95** | **95** | **96** | **96** | **97** |

*Supplementary Table 6*

| **Plasma Concentration [ng/mL]** | | **Olanzapine** | | | | | | |
| --- | --- | --- | --- | --- | --- | --- | --- | --- |
|  |  | **0** | **10** | **20** | **30** | **40** | **60** | **100** |
| **Aripiprazole** | **0** | **0** | **59** | **74** | **81** | **85** | **90** | **93** |
|  | **50** | **83** | **87** | **89** | **90** | **91** | **93** | **95** |
|  | **100** | **91** | **92** | **93** | **93** | **94** | **95** | **96** |
|  | **200** | **95** | **96** | **96** | **96** | **96** | **97** | **97** |
|  | **300** | **97** | **97** | **97** | **97** | **97** | **97** | **98** |
|  | **500** | **98** | **98** | **98** | **98** | **98** | **98** | **98** |
|  | **1000** | **99** | **99** | **99** | **99** | **99** | **99** | **99** |

*Supplementary Table 7*

| **Plasma Concentration [ng/mL]** | | **Olanzapine** | | | | | | |
| --- | --- | --- | --- | --- | --- | --- | --- | --- |
|  |  | **0** | **10** | **20** | **30** | **40** | **60** | **100** |
| **Haloperidol** | **0** | **0** | **59** | **74** | **81** | **85** | **90** | **93** |
|  | **1** | **76** | **82** | **86** | **88** | **90** | **92** | **95** |
|  | **2** | **86** | **88** | **90** | **91** | **92** | **94** | **95** |
|  | **3** | **90** | **92** | **92** | **93** | **94** | **95** | **96** |
|  | **5** | **94** | **94** | **95** | **95** | **96** | **96** | **97** |
|  | **10** | **97** | **97** | **97** | **97** | **97** | **98** | **98** |
|  | **15** | **98** | **98** | **98** | **98** | **98** | **98** | **98** |

*Supplementary Table 8*

| **Plasma Concentration [ng/mL]** | | **Haloperidol** | | | | | | |
| --- | --- | --- | --- | --- | --- | --- | --- | --- |
|  |  | **0** | **1** | **2** | **3** | **5** | **10** | **15** |
| **Aripiprazole** | **0** | **0** | **76** | **86** | **90** | **94** | **97** | **98** |
|  | **50** | **83** | **89** | **92** | **93** | **95** | **97** | **98** |
|  | **100** | **91** | **93** | **94** | **95** | **96** | **98** | **98** |
|  | **200** | **95** | **96** | **96** | **97** | **97** | **98** | **99** |
|  | **300** | **97** | **97** | **97** | **98** | **98** | **98** | **99** |
|  | **500** | **98** | **98** | **98** | **98** | **98** | **99** | **99** |
|  | **1000** | **99** | **99** | **99** | **99** | **99** | **99** | **99** |

*Supplementary Table 9*

| **Plasma Concentration [ng/ml]** | | **Risperidone** | | | | | | |
| --- | --- | --- | --- | --- | --- | --- | --- | --- |
|  |  | **0** | **10** | **20** | **30** | **40** | **60** | **100** |
| **Aripiprazole** | **0** | **0** | **59** | **74** | **81** | **85** | **90** | **93** |
|  | **50** | **83** | **87** | **89** | **90** | **91** | **93** | **95** |
|  | **100** | **91** | **92** | **93** | **93** | **94** | **95** | **96** |
|  | **200** | **95** | **96** | **96** | **96** | **96** | **97** | **97** |
|  | **300** | **97** | **97** | **97** | **97** | **97** | **97** | **98** |
|  | **500** | **98** | **98** | **98** | **98** | **98** | **98** | **98** |
|  | **1000** | **99** | **99** | **99** | **99** | **99** | **99** | **99** |

*Supplementary Table 10*

| **Plasma**  **Concentration [ng/mL]** | | **Risperidone** | | | | | | |
| --- | --- | --- | --- | --- | --- | --- | --- | --- |
|  |  | **0** | **10** | **20** | **30** | **40** | **60** | **100** |
| **Haloperidol** | **0** | **0** | **67** | **80** | **86** | **89** | **92** | **95** |
|  | **1** | **76** | **84** | **88** | **90** | **92** | **94** | **96** |
|  | **2** | **86** | **89** | **91** | **92** | **93** | **95** | **96** |
|  | **3** | **90** | **92** | **93** | **94** | **95** | **96** | **97** |
|  | **5** | **94** | **95** | **95** | **96** | **96** | **97** | **97** |
|  | **10** | **97** | **97** | **97** | **97** | **98** | **98** | **98** |
|  | **15** | **98** | **98** | **98** | **98** | **98** | **98** | **99** |

We also evaluated our model using different EC50 values for clozapine. Specifically, we applied an EC50 of 950 ng/mL, as determined by Gründer et al. (1) for the putamen (*Supplementary Table 11 – 14*). The resulting receptor occupancy percentages showed only minor differences compared to those calculated using the EC50 value proposed by Uchida et al. (2) (*Supplementary Table 1 – 4*).

*Supplementary Table 11*

| **Serum Concentration [ng/mL]** | | **Haloperidol** | | | | | | |
| --- | --- | --- | --- | --- | --- | --- | --- | --- |
|  |  | **0** | **1** | **2** | **3** | **5** | **10** | **15** |
| **Clozapine** | **0** | **0** | **76** | **86** | **90** | **94** | **97** | **98** |
|  | **200** | **17** | **77** | **87** | **91** | **94** | **97** | **98** |
|  | **400** | **30** | **78** | **87** | **91** | **94** | **97** | **98** |
|  | **600** | **39** | **79** | **87** | **91** | **94** | **97** | **98** |
|  | **800** | **46** | **80** | **88** | **91** | **94** | **97** | **98** |
|  | **1000** | **51** | **81** | **88** | **91** | **94** | **97** | **98** |
|  | **1200** | **56** | **81** | **88** | **91** | **94** | **97** | **98** |

*Supplementary Table 12*

| **Serum Concentration [ng/ml]** | | **Aripiprazole** | | | | | | |
| --- | --- | --- | --- | --- | --- | --- | --- | --- |
|  |  | **0** | **50** | **100** | **200** | **300** | **500** | **1000** |
| **Clozapine** | **0** | **0** | **83** | **90** | **95** | **97** | **98** | **99** |
|  | **200** | **17** | **84** | **91** | **95** | **97** | **98** | **99** |
|  | **400** | **30** | **84** | **91** | **95** | **97** | **98** | **99** |
|  | **600** | **39** | **85** | **91** | **95** | **97** | **98** | **99** |
|  | **800** | **46** | **85** | **92** | **95** | **97** | **98** | **99** |
|  | **1000** | **51** | **86** | **92** | **95** | **97** | **98** | **99** |
|  | **1200** | **56** | **86** | **92** | **96** | **97** | **98** | **99** |

*Supplementary Table 13*

| **Serum Concentration [ng/mL]** | | **Olanzapine** | | | | | | |
| --- | --- | --- | --- | --- | --- | --- | --- | --- |
|  |  | **0** | **10** | **20** | **30** | **40** | **60** | **100** |
| **Clozapine** | **0** | **0** | **59** | **74** | **81** | **85** | **90** | **93** |
|  | **200** | **17** | **62** | **75** | **82** | **86** | **90** | **94** |
|  | **400** | **30** | **65** | **77** | **82** | **86** | **90** | **94** |
|  | **600** | **39** | **67** | **78** | **83** | **86** | **90** | **94** |
|  | **800** | **46** | **69** | **79** | **84** | **87** | **90** | **94** |
|  | **1000** | **51** | **71** | **80** | **84** | **87** | **91** | **94** |
|  | **1200** | **56** | **73** | **80** | **85** | **87** | **91** | **94** |

*Supplementary Table 14*

| **Serum Concentration [ng/mL]** | | **Risperidone** | | | | | | |
| --- | --- | --- | --- | --- | --- | --- | --- | --- |
|  |  | **0** | **10** | **20** | **30** | **40** | **60** | **100** |
| **Clozapine** | **0** | **0** | **67** | **80** | **86** | **89** | **92** | **95** |
|  | **200** | **17** | **69** | **81** | **86** | **89** | **92** | **95** |
|  | **400** | **30** | **71** | **82** | **87** | **89** | **93** | **95** |
|  | **600** | **39** | **72** | **82** | **87** | **90** | **93** | **95** |
|  | **800** | **46** | **74** | **83** | **87** | **90** | **93** | **95** |
|  | **1000** | **51** | **75** | **83** | **88** | **90** | **93** | **95** |
|  | **1200** | **56** | **77** | **84** | **88** | **90** | **93** | **96** |

Only one study reports estimates for EC_50_ that are based on active moiety (aripiprazole + dehydroaripiprazole) concentrations of the drug (3). Results are in accordance with the therapeutic reference range of the active moiety (150 – 500 ng/ml) (4), implying that higher levels are necessary to surpass the necessary receptor occupancy threshold. We have carried out calculations with active moiety and included them in the supplement.

*Supplementary Table 15*

| **Plasma Concentration [ng/ml]** | | **Aripiprazole (Aripiprazole + Dehydroaripiprazole)** | | | | | | |
| --- | --- | --- | --- | --- | --- | --- | --- | --- |
|  |  | **0** | **50** | **100** | **200** | **300** | **500** | **1000** |
| **Clozapine** | **0** | **0** | **71** | **83** | **91** | **94** | **96** | **98** |
|  | **200** | **29** | **74** | **84** | **91** | **94** | **96** | **98** |
|  | **400** | **45** | **77** | **85** | **92** | **94** | **96** | **98** |
|  | **600** | **55** | **79** | **86** | **92** | **94** | **96** | **98** |
|  | **800** | **62** | **81** | **87** | **92** | **94** | **96** | **98** |
|  | **1000** | **67** | **82** | **88** | **93** | **94** | **96** | **98** |
|  | **1200** | **71** | **83** | **88** | **93** | **95** | **96** | **98** |

1. **Supplementary Calculations**

**
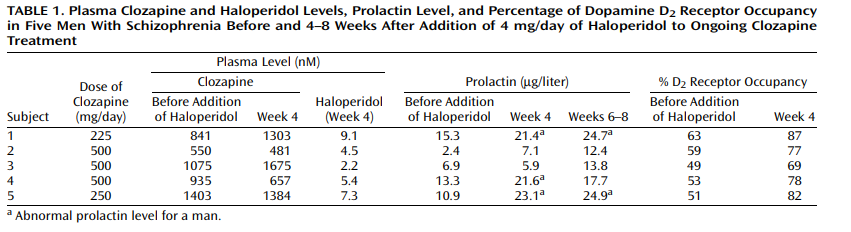
**

*From (5)*

To enable comparison of model estimates with values of Kapur et al., we convert plasma levels (nmol/l) to ng/ml, using the corresponding molecular weights: clozapine (326.8 g/mol) and haloperidol (375.9 g/mol).

**Nmol/L = (ng/mL * MW) / 1000 [1]**

**
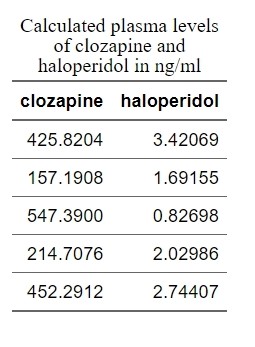
**

After this step, we include the respective plasma levels using he EC_50_ values noted above (0.32 ng/ml for haloperidol, 483 ng/ml for clozapine).

**occupancy [%] = 100 – 100 / (1 + C1/EC_50_1 + C2/EC_50_2) [2]**

| **Clozapine level**  **(in ng/ml)** | **Haloperidol level**  **(in ng/ml)** | **Receptor occupancy (estimated)**  **(in %)** | **Receptor occupancy (measured)**  **(in %)** | **Absolute difference**  **(in %)** |
| --- | --- | --- | --- | --- |
| **426** | **3.4** | **92** | **87** | **5** |
| **157** | **1.7** | **85** | **77** | **8** |
| **547** | **0.8** | **78** | **69** | **9** |
| **214** | **2.0** | **87** | **78** | **9** |
| **452** | **2.7** | **90** | **82** | **8** |

In our model, we see a mean + standard deviation (SD) absolute difference of 7-8 ± 1.6 % (5 - 9 %) between the estimated striatal D_2_R occupancy at the indicated plasma concentrations and the measured D_2_R occupancy to PET. The observed and predicted values of D_2_Roccupancy showed a high correlation (*r* = 0.98, *P* < 0.005). The magnitude of the absolute differences is similar to that for the individual substances, namely 12.1 ± 9.1 % for clozapine and 7.0 ± 7.1 % for haloperidol. The mean prediction error is also 7.8 %, because all predicted values are higher than the measured values in PET.

1. **Literature References of the SI**

1. Gründer G, Landvogt C, Vernaleken I, Buchholz HG, Ondracek J, Siessmeier T, et al. The striatal and extrastriatal D2/D3 receptor-binding profile of clozapine in patients with schizophrenia. *Neuropsychopharmacology*. 2006;**31**(5):1027-35.

2. Uchida H, Takeuchi H, Graff-Guerrero A, Suzuki T, Watanabe K, Mamo DC. Predicting dopamine D₂ receptor occupancy from plasma levels of antipsychotic drugs: a systematic review and pooled analysis. *J Clin Psychopharmacol*. 2011;**31**(3):318-25.

3. Gründer G, Fellows C, Janouschek H, Veselinovic T, Boy C, Bröcheler A, et al. Brain and plasma pharmacokinetics of aripiprazole in patients with schizophrenia: an [18F]fallypride PET study. *Am J Psychiatry*. 2008;**165**(8):988-95.

4. Hiemke C, Bergemann N, Clement HW, Conca A, Deckert J, Domschke K, et al. Consensus Guidelines for Therapeutic Drug Monitoring in Neuropsychopharmacology: Update 2017. *Pharmacopsychiatry*. 2018;**51**(1-02):9-62.

5. Kapur S, Roy P, Daskalakis J, Remington G, Zipursky R. Increased dopamine d(2) receptor occupancy and elevated prolactin level associated with addition of haloperidol to clozapine. *Am J Psychiatry*. 2001;**158**(2):311-4.
